# Permeate Flux in Ultrafiltration Processes—Understandings and Misunderstandings

**DOI:** 10.3390/membranes12020187

**Published:** 2022-02-05

**Authors:** Robert W. Field, Jun Jie Wu

**Affiliations:** 1 Faculty of Engineering and Environment, Northumbria University, Newcastle NE1 8ST, UK; 2 Department of Engineering Science, University of Oxford, Oxford OX1 3PJ, UK; 3 Department of Engineering, Faculty of Science, Durham University, Durham DH1 3LE, UK

**Keywords:** concentration polarization, osmotic model, fouling, modes of fouling, design

## Abstract

Concentration polarization refers to the rapid emergence of concentration gradients at a membrane/solution interface resulting from selective transfer through the membrane. It is distinguishable from fouling in at least two ways: (1) the state of the molecules involved (in solution for concentration polarization, although no longer in solution for fouling); and (2) by the timescale, normally less than a minute for concentration polarization, although generally at least two or more orders of magnitude more for fouling. Thus the phenomenon of flux decline occurring over a timescale of tens of minutes should not be attributed to concentration polarization establishing itself. This distinction and a number of questions surrounding modelling are addressed and clarified. There are two paradigmatic approaches for modelling flux, one uses the overall driving force (in which case allowance for osmotic effects are expressed as additional resistances) and the other uses the net driving force across the separating layer or fouled separating layer, although often the two are unfortunately comingled. In the discussion of flux decline models’ robust approaches for the determination of flux-time relationships, including the integral method of fouling analysis, are discussed and various concepts clarified. The final section emphases that for design purposes, pilot plant data are vital.

## 1. Introduction

It is our privilege to have received mentorship from and been befriended by Tony over what is now an extended period. In the case of one of us, this stretches back to one of his research trips to Europe over three decades ago (RWF), and for the rest of us, to two decades ago when one of us (JJW) was a Tan Chin Tuan academic exchange fellow at Nanyang Technological University. In bearing witness to the fact that his insatiable passion for membrane research is still very much alive, mention is made of his insightful contribution to a very recent paper we co-authored comparing fouling of a thin-film composite membrane in reverse osmosis mode with the corresponding behavior in forward osmosis mode [1]. This is about 40 years since his first contributions to the Journal of Membrane Science and to Desalination, though not his first contributions to membrane science [2,3] These early papers were, respectively, on membrane surface pore characteristics and flux through ultrafiltration membranes, and factors affecting flux in crossflow filtration subjects, which are of concern herein.

His international recognition is in part due to his prolific published output but also to his editorship of the Journal of Membrane Science and to his contributions to conferences both large and small. The latter category includes the long series of roughly biennial Oxford workshops on membranes and water treatment starting with one on critical flux in 2002 and including the first UK–Israeli Workshop and Research Event on the Application of Membrane Technology in Water Treatment and Desalination in 2008, one on Membrane (Bio)Fouling in 2012 and one on Recent Advances in Membrane Science and Technology in 2015. At these workshops and when giving plenary lectures he brought great clarity to the various concepts under discussion.

As fellow academics, whose formation was also chemical engineering, we note that we share similar concerns. At the heart of chemical and process engineering is the design and operation of processes that change the thermodynamic state, composition, morphology or other characteristic of materials. Thus it is fitting to include a section on the design of membrane systems. Herein we confine ourselves to membrane separations and thus the concern is with a change in composition with a feed stream being separated into two streams one that has passed through the membrane and one that has been retained above it. Although a number of basic issues are addressed below, all are linked to the ultimate aims of understanding and of improving a separation process, in which a desired degree of concentration or purification is achieved at a quantifiable rate propelled by a known driving force.

The format of this paper is a little unusual and has been designed with the aim of bringing clarity and insight to a number of areas. The laudably high aim is to achieve a similar level of clarity to that demonstrated by Tony over the past 40+ years and counting. This is a clarity that is often regrettably missing in the huge proliferation of papers today. Each of the subsequent sub-sections start with a question and these are grouped under one of three principal sections on Fundamentals, Modelling and Design. Some of these questions reflect subtleties and some might, by some readers, be seen as basic tutorial questions. The common thread is that all have arisen from us observing misunderstandings and over-simplifications in the recent peer-reviewed literature.

## 2. Fundamentals

### 2.1. Is Concentration Polarisation Distinct from Fouling?

Concentration polarization refers to the emergence of concentration gradients at a membrane/solution interface resulting from selective transfer of some species through the membrane. The link between this natural consequence of permselectivity and the attenuation of driving forces across the active layer of the membranes themselves was explored in some detail for a range of membrane processes previously [4]. Here it is simply emphasized, paraphrasing the often-overlooked work of Aimar et al. [5] that fouling and concentration polarization effects are distinguishable by two facts: (1) the state of the molecules involved (in solution concentration polarization, although no longer in solution for fouling); and (2) by the timescale, normally less than a minute for concentration polarization, although generally at least two or more orders of magnitude for fouling. They established the timescales through elegant experimentation involving the measurement of optical density. The response curves indicated that the build-up of the concentration polarization was achieved within 30 s in all of their experiments, which as they noted is a value that accords with various theoretical calculations [6]. The experiments just referred to involved bovine serum albumin and for all macromolecules it is readily appreciated that there is a clear distinction between the build-up of macromolecules that remain in solution adjacent to an interface and the build-up of macromolecules no longer in solution that are deposited at the interface.

Thus to the question posed above, namely; *Is concentration polarization distinct from fouling?* The answer to the ultrafiltration of macromolecules must undoubtedly be “yes”. Indeed if researchers are to use the term, ‘concentration polarization’ in a manner consistent with the IUPAC definition that it refers to the “concentration profile that has a higher level of solute nearest to the upstream membrane surface compared with the more-or-less well- mixed bulk fluid far from the membrane surface” [7], it is absolutely clear that it has nothing to do with fouling. This is absolutely consistent with the statement made by researchers from another laboratory [8] who distinguished between “(i) concentration polarization (i.e., accumulation of retained solutes, reversibly and immediately occurring) and (ii) fouling phenomena such as adsorption, pore-blocking and deposition of solidified solutes, a long-term, and more or less irreversible process.”

**Figure 1:**
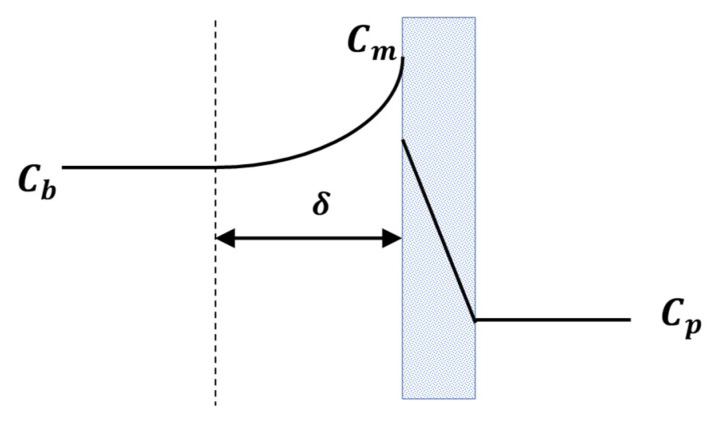
Schematic of concentration polarization phenomena in ultrafiltration. Between the bulk feed solution (the region of constant $\;C_{b}$) and the membrane there is the mass transfer boundary layer of thickness δ. Within this region the concentration is said to be polarized.

Whilst maintaining a distinction between concentration and fouling, some authors such as those who write, “Concentration polarization (CP), produced by the accumulation of solutes on the membrane surface, causes an increased resistance to solvent transport and possibly a change in the separation characteristics of the membrane” [9] clearly create room for a number of subtle misunderstandings. Firstly there should be no change to the intrinsic separation characteristics of the membrane as the pores have not been blocked or even partially blocked by CP and secondly there is no accumulation of solutes ***on*** the membrane surface; CP refers to the mass transfer layer adjacent to the upstream membrane surface and the associated equation includes the thickness of this mass transfer layer: As shown elsewhere (e.g., [10,11]) the relationship linking the concentrations of the solute in the bulk ($C_{b}$), at the interface with the membrane ($C_{m}$) and in the permeate ($C_{p}$) is:

(1) $$C_{m}=\left(C_{b}-C_{p}\right)\exp\left(Pe\right)+C_{p}$$

where Pe is the boundary layer Peclet number and is defined as Jδ/D. *J* is the volumetric flux through the membrane, δ is the thickness of the mass transfer boundary layer and *D* is the diffusivity of the solute. The relationship is shown schematically in Figure 1.

### 2.2. Is Concentration Polarisation the Primary Reason for Flux Decline during the Initial Period of Operation?

The quotation from a recent 2021 paper given towards the end of the previous section continues, “CP is considered as a reversible phenomenon and is the primary reason of flux decline during the initial period of operation” [9]. Elsewhere it is not uncommon for authors to mistakenly attribute the flux decline over the first 10–30 min to CP. See for example Qaid et al. [12], who in their discussion of their Figures 4 and 5 in [12] attribute all of the flux decline in the initial 25 min to concentration polarization, and only the much more modest slight decline thereon is attributed to fouling. Whilst CP can lead to a reduction in driving force, as discussed later in Section 2.5, this reduction, will be rapid. Therefore the aforementioned reduction over 25 min has to be attributed, in part, to other causes. The reductions observed by Qaid et al. [12], with their two membranes, during ultrafiltration of orange juice, would not have been due to CP *per se.* CP leads to higher concentration at the membrane surface as indicated by Equation (1) and this can exacerbate the formation of a fouling layer, although such a fouling layer is not reversible. 

We concur with [9] that CP is a reversible phenomenon. Indeed it is suggested that the statement “CP is considered as a reversible phenomenon” be strengthened so as to state that CP ***is*** a reversible phenomenon. The answer that we would posit to the question *Is concentration polarisation the primary reason for flux decline during the initial period of operation?* is this: if you are referring to an initial period of up to a few minutes then the answer is ‘maybe’, however if you are referring to a period beyond that then the principal reason for flux decline will be due to other factors.

### 2.3. Can Gel Theory Explain Flux Decline?

To many, this will seem a strange question as in the twentieth century gel theory was never concerned with the time evolution of fouling. However the reason for raising this question will become apparent shortly. Firstly, we rearrange Equation (1) to obtain an expression for the *limiting* flux at a surface concentration that is equal to a gel concentration i.e., $C_{m}=C_{gel}$. If this concentration is fixed (and in the gel-polarisation model it is considered fixed) then from Equation (1), one can readily derive Equation (2). Clearly for a given value of bulk concentration and fixed mass transfer conditions, the flux is predicted to have a unique value, the limiting flux, $J_{lim}$, as given by:

(2) $$J_{lim}=k.\;\ln\;\left(\frac{C_{gel}-C_{p}}{C_{b}-C_{p}}\right);\;k=D/\delta$$

For cross-flow ultrafiltration the attraction of gel-polarization theory [8] was that it could explain many experimental results such as (i) flux approximately proportional to $-\ln\left(C_{b}\right)$, (ii) flux approximate proportional to mass transfer coefficient, and (iii) in an age when the tendency was to maximize flux and work in the plateau region, a reason for the insensitivity of flux to applied pressure. However, the assumption that the concentration at the membrane interface cannot exceed a fixed concentration leads to a peculiar assumption namely that “an increase in the applied pressure will then only result in an increased thickness in the gel layer, although not in an increase in flux”. That non-gelling macromolecules also displayed the same experimental behavior of flux being approximately proportional to $-\ln\left(C_{b}\right)$ and to mass transfer coefficient, k, coupled with the characteristic plateauing of flux at high TMP led to the emergence of other theories as discussed elsewhere [8,13,14]. Also gel-polarisation theory could not and cannot explain why for one given solute, the limiting concentration determined from a plot of $J_{lim}$ versus $-\ln\left(C_{b}\right)$ as the point where flux would go to zero, varies when that solute is filtered in two different filtration-cells [8,15]. Also as pointed out by others the supposedly constant value of $C_{gel}$. seems to vary during filtration [9,16,17].

It will be apparent that the answer to the question, *Can gel theory explain flux decline*, is, for crossflow, filtration “no”. In a batch system one can envisage an increasing bulk concentration with a consequential decrease in the mass transfer coefficient and thus for these reasons a decrease in the flux. However, such a decrease in flux would not be due to the evolution of a fouling layer (although the two changes could occur side-by-side). The reason for positing the question is that equations in some recent publications (e.g., see Equation (2) in [9]), are identical to Equation (2) above, except the subscript is omitted from the flux term and additionally the accompanying text treats *J* as a general term that gives the clear impression that it could be a shorthand for J(t). From those who originated gel-polarization theory it is clear that the predicted flux is the plateau flux and this should always be made clear.

As the performance of membrane processes are strongly influenced by the accumulation of retained solutes at the membrane surface, concentration polarization needs to be taken into account. Thus film theory is highly relevant but the general adoption of the concept of gel-polarization is not only necessary and is often a distraction. “Film theory” is the application of Equation (1). Rewriting it as Equation (3) it is simply expressing a link between the bulk concentration, the concentration at the solid-fluid interface and flux.

(3) $$J=k.\;\ln\;\left(\frac{C_{m}-C_{p}}{C_{b}-C_{p}}\right);\;k=D/\delta$$

In ultrafiltration the increase in the concentration of macromolecules at the interface will increase the osmotic pressure difference across the membrane and lead to viscosity increases in the boundary layer, which decrease the mass transfer coefficient, principally by increasing the value of the boundary layer thickness, δ. These factors are explored in the following sections.

### 2.4. Can the Effect of Viscosity upon Mass Transfer Explain a Limiting Flux?

The boundary layer thickness  δ is not the value that would be obtained under isoviscous conditions i.e., conditions where there is no change in viscosity across the boundary layer and this was shown to be a crucial factor [14]. It might be argued that an exclusive focus upon viscosity ignores changes in density and diffusivity, however Fane and others [18] concluded that an examination of physical property data shows: (a) the variations in density are negligible; and (b) for the systems such as BSA, sucrose and dextran the variations in viscosity are much larger than those in diffusivity. They posed the question “How does the viscosity variation with concentration affect the level of polarisation?” and in answering this question they noted that a “blow-up” in polarization can occur when the variation in viscosity with concentration is considered.

So in looking at the concentration dependency of k (=D/δ), it is considered reasonable to relate the reduction in mass transfer coefficient solely to a viscosity ratio. This is not offered as a general observation on the cause of flux reduction as osmotic pressure changes will also influence the overall driving force as discussed in the next section. Additionally, for most solutions (but not all) there will the potential of fouling. Nevertheless, the effect of macroscopic viscosity upon the velocity profile has been shown to be significant and the viscosity dependency of the mass transfer coefficient can on its own in principle lead to a limiting flux [19]. So the answer to the question posed is ‘yes’. Whilst the viscosity profile is more important than physiochemical phenomena such as changing solute mobilities, an equally important phenomenon is the increase in osmotic pressure at the interface due to the concentration profile.

### 2.5. Can the Effect of Osmotic Pressure Explain a Limiting Flux?

Concentration polarization has four main effects [14]: (a) changes in the physico-chemical properties of the fluid within the membrane boundary layer (e.g., viscosity); (b) an enhanced osmotic pressure difference across the membrane that partially offsets the applied pressure difference; (c) changes in the membrane properties due to membrane-solute interactions (i.e., fouling); and (d) the potential of gelation at sufficiently high surface concentrations of certain macromolecules. Thus osmotic pressure models are intrinsically linked to the phenomenon of concentration polarization. 

Aimar and Sanchez [20] quantitatively linked effects (a) and (b). They proposed a model to describe the relationship between permeate flux,  J, TMP, $C_{b}$ and crossflow velocity for a range of fluxes from zero to the limiting value with a limited number of parameters being obtained from experimental data. Their model, like all other osmotic pressure models, did not predict the slight decrease in flux, which can occur at high transmembrane pressures. Now for solutions that do not foul or gel at high concentrations (e.g., those of dextran) there is some evidence that at high pressures and low crossflow velocities the flux decreases slightly with increasing pressure. Later it was confirmed from a theoretical viewpoint that such a decrease can only be caused by concentration increases in the boundary layer leading to a decrease in the average mass-transfer coefficient [14]. These authors also proposed a methodology for the calculation of permeate fluxes as a function of the transmembrane pressure for the ultrafiltration of ideal solutions, where ‘ideal’ indicates the absence of fouling and gelation. The model allows for the influence of both osmotic pressure and the variation in viscosity due to concentration polarization. Thus to make predictions knowledge of osmotic pressure and viscosity as a function of solute concentration is required. The only membrane parameter that has to be experimentally determined is the membrane permeability. The set of equations that need to be solved are the following pair of equations, one of which (Equations (3)) has already been introduced:

(3) $$J=k.\;\ln\;\left(\frac{C_{m}-C_{p}}{C_{b}-C_{p}}\right)$$

(4) $$J=\frac{\Delta P-\Delta \pi }{\mu_{p}R_{m}}$$

where $\mu_{p}$ is the viscosity of the permeate and $R_{m}$ is the hydraulic resistance of the membrane, ΔP is the TMP across the membrane and Δπ is the osmotic pressure difference across the membrane itself. Often (e.g., as in [9]) this is not made clear. To be precise, the difference $\Delta \pi =\pi_{m}-\pi_{p}$ where the terms on the righthand side are, respectively, the osmotic pressure *at the interface* on the upstream side and on the permeate side. To calculate the osmotic pressure difference a relationship between osmotic pressure and solute concentration is required. For many macromolecular solutions an appropriate expression takes the form of a virial expansion. Thus,

(5) $$\pi =B_{1}C+B_{2}C^{2}+B_{3}C^{3}$$

where *B*_1_, *B*_2_, and *B*_3_ are the osmotic virial coefficients, and *C* is the concentration of the macromolecular solute (g L^−1^). 

An expression similar to Equation (5) above is required for the viscosity of the feed solution. Given this information the mass transfer, *k* can be linked to that which would hold for isoviscous conditions. The correction factor adopted in [14] was similar to that used by others in the field of ultrafiltration [21,22]. The expression for the mass transfer coefficient, *k* is then given by:

(6) $$k=k_{0}{\left(\mu_{b}/\mu_{m}\right)}^{z}$$

where $k_{0}$ is the mass transfer coefficient for isoviscous conditions and the viscosity terms refer to the viscosity of the bulk solution and to that of the concentrated solution at the membrane surface. The index *z* will have a value of around 0.14 [14,23]. 

Thus the osmotic pressure model (preferably with due allowance for the effect of boundary layer viscosity gradient upon mass transfer) accomplishes everything that the gel-polarization model achieved and more. There is no need to hypothesize a gel layer and the full range of flux is covered.

### 2.6. What Is the Flux Paradox?

When filtering colloidal suspensions “puzzlingly high” fluxes can be observed; membranologists refer to these as “puzzlingly high” as Equation (3) indicates the flux should decrease with decreasing values of diffusivity, *D*. Now larger particles have lower values of *D*, as given by calculations of Brownian diffusion, than macromolecules, however the observed fluxes are higher. Hence, the use of the term ‘paradox’.

One of the first observations of this “colloid flux paradox” can be attributed to Cohen et al. [24]. A physical explanation for it can be acquired through balancing surface interaction, diffusion and convection [25]. When compared to other transport phenomena, surface interaction has been shown, for particle size between 10 nanometers and 10 microns to be responsible for fluxes, which are well above the ones given with other transport phenomena such as diffusion, shear induced diffusion and lateral migration. The potential importance of surface interactions is well illustrated elsewhere, see for example Figure 3 in [26]. In the absence of significant charge interactions, the dominant mechanism will depend on the particle size and the tangential shear rate. As noted in [9], “for typical shear rates, Brownian diffusion is important for submicron particles, the inertial lift is important for particles larger than approximately 10 microns, and shear-induced diffusion is dominant for intermediate-sized particles.” The equations relevant to these mechanisms can be found in [27] and for an overall review see [28]. In terms of the equations given above, one can say that the back-transport mechanisms will influence the “*k*” in Equation (3). Membrane permeability does have a direct effect upon the mechanisms governing diffusion, however the link between Equations (3) and (4) is the overall flux and this is determined in part by the transmembrane pressure and the membrane resistance. Predictions for complex mixtures involving colloids are extremely problematic and further comment is left until Section 4.

## 3. Modelling of Flux Decline

### 3.1. What Is an Appropriate Classification for Flux Decline Models?

A statement such as “Some authors [five references given] have said that the models applied in UF for flux prediction can be grouped into five categories: (i) concentration polarization models; (ii) osmotic pressure models; (iii) resistance-in-series models; (iv) fouling models, based on the classical film theory model; and (v) non-phenomenological models” [9] will puzzle many. Questions arising ask whether there is an agreed position in the literature that there are five category of models, and, whether there five distinct categories. Upon consulting the first of the five references (e.g., [8] which was mentioned in another context above) it is clear that ‘five’ is not an agreed position. That 1990 paper actually mentions just three categories almost corresponding to (i) to (iii) however instead of ‘concentration polarization’ they mention ‘gel-polarization’, which as discussed in Section 2.3 (and captured by Equations (2) and (3)) are two related yet distinct concepts. Confusingly, Figure 1 of [9] is inconsistent with the text that was quoted at the beginning of the paragraph. 

The number of categories should follow from a careful consideration of their underlying basis. Now from the discussion in Section 2 it will be clear that concentration polarization is part of the foundation of osmotic models. Therefore the separating off of osmotic models can lead to a false dichotomy. Clearly the creation of a division runs the risk of creating a false understanding that they are not linked. Likewise, the separation of resistance-in-series models from fouling models ignores the fact that a fouling model based upon cake formation is a model that has a cake resistance in series with a membrane resistance. Hermia’s seminal paper [29] on constant pressure filtration, which they include in their fourth category, has nothing to do with “classical film theory” and thus the qualification of the category “(iv) fouling models” by the addition of the phrase, “based on the classical film theory model” is not illuminating and instead confuses. An appropriate classification would distinguish between models that generate expressions for ideal fluxes (i.e., no fouling included) and those that include fouling. With regard to fouling models, there are those models that allow for length of the flow channels (see example in [27]) and those that do not [29,30]. In agreeing with [27] that for short times the transient flux decline is closely approximated by dead-end batch filtration theory, it is noted that the qualification ‘short’ is important. Thus in accordance with others e.g., [27,30] the steady or quasi-steady flux at longer times will only be appropriately modelled by expressions that include a term that can reflect the steady or quasi steady flux. Thus it is a category mistake to treat dead-end and crossflow expressions as equivalent (as for example in [31]) and an appropriate taxonomy of UF and MF models should reflect their difference. Logicians would say that a category mistake has been committed when, once the phenomenon in question is properly understood, it becomes clear that the claim being made could not possibly be true. To us the appropriate number of categories of fouling models is a matter for debate, however the misunderstanding that one can analyze, without qualification, crossflow experimental data using dead-end models is a category mistake that needs to be avoided.

### 3.2. What Is the Appropriate Viscosity in the Resistance-In-Series Model? 

Now, we generalize the equation in Section 2.5 to allow for fouling:

(7) $$J=\frac{\Delta P-\Delta \pi }{\mu_{p}R_{t}};\;\;\;\;\;\;R_{t}=R_{m}+R_{f}$$

where $\mu_{p}$ is the viscosity of the permeate and $R_{t}$ is the overall resistance $R_{f}$ is the resistance due to fouling. It is reiterated that the relevant viscosity is that of the permeate. Often (e.g., [9,31] there is just a reference to “the viscosity of solution” and the viscosity term is labelled simply as μ. When this is coupled with a mention that viscosity increases with solute concentration, there is the clear impression that the relevant viscosity is that of the feed solution rather than that of the permeate. Indeed, in some papers the viscosity used in the calculation of resistance (e.g., Equation (15) in [32]) is clearly stated to be that of the feed, which is erroneous for the following reason. The permeate is passing through the membrane and thus, to be consistent with Darcy’s law the relevant viscosity should be that of the permeate, as clearly set out by the majority of researchers, e.g., [8,27,33].

### 3.3. When Is It Appropriate to Include a Concentration Polarisation Resistance Term?

In Equation (7) the driving force was that across the membrane itself, ΔP−Δπ. An alternative to this equation is to relate the flux to the overall driving force between the *bulk* fluid on one side and *bulk* fluid on the other side. With this approach one has to ascribe a resistance to the concentration polarization layer, $R_{cp}$, and this gives, with inclusion of a separate fouling resistance term:

(8) $$J=\frac{\Delta P}{\mu_{p}R_{t}};\;\;\;\;\;\;\;\;R_{t}=R_{m}+R_{cp}+R_{f}$$

As others [34,35] have shown that the expressions in Equations (7) and (8) are thermodynamically equivalent the answer to the question, *When is it appropriate to include a concentration polarization resistance term* is as follows: it is appropriate to include a resistance to the concentration polarization layer, $R_{cp}$ in the denominator provided the driving force is from the bulk feed on one side to the permeate on the other. One can consider that the concentration boundary layer impedes the flow of the solvent and thus “consumes” part of the overall driving force. If this approach is taken, then Equation (8) is used. A common misunderstanding is to pair the numerator of Equation (7) with the denominator of Equation (8), however this mistakenly double counts the effect of the boundary layer. 

### 3.4. Is There a Robust Methodology for Fouling Analysis of Crossflow Systems?

**Figure 2:**
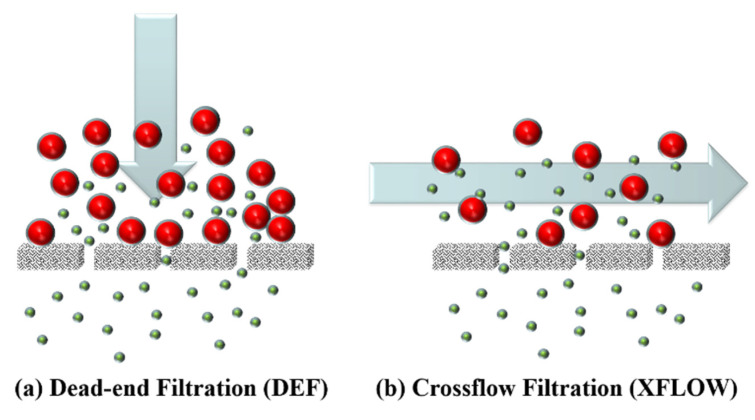
Main flow types in membrane separations: (**a**) Dead-end filtration; and (**b**) Crossflow filtration. Large (red) spheres represent rejected particles, small (green) spheres represent non-rejected particles, and the arrows indicate the feed flow direction. Reproduced with permission from Elsevier.

The main question is prefaced by another: what is the attraction of dead-end filtration models? A tentative answer to this question might be threefold and reference the attraction of deceptive simplicity, perhaps mention that Equation (9) below is intriguing and finally touch upon the fact that a straightforward methodology of fouling analysis, with due allowance for crossflow, has yet to establish itself. As illustrated in Figure 2, crossflow inhibits the build-up of particles and thus the associated mass balances the need to have a removal term as discussed elsewhere [30,36]. The allowance for crossflow leads to a modest increase in the complexity of fouling analysis, however it is a necessity to accept this complexity. Even for early times, a check should be made to see whether use of dead-end analysis is appropriate or not.

**Figure 3:**
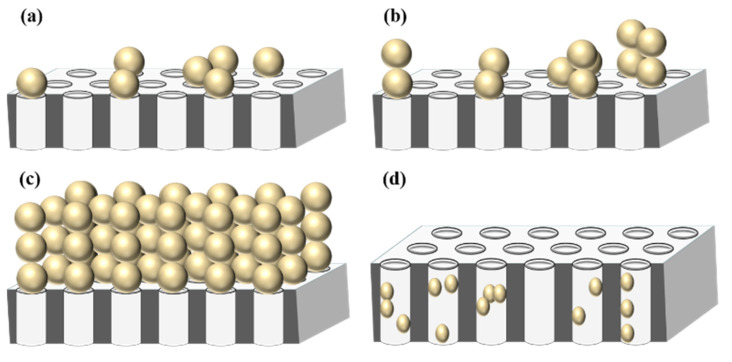
Schematic representation of Hermia’s fouling mechanisms: (**a**) Complete pore blocking; (**b**) Intermediate pore blocking; (**c**) Cake filtration; and (**d**) Pore filling. The values of the fouling index *n* associated with mechanisms (**a**–**d**) are, respectively, 2, 1, 0 and 1.5. Adsorption is not included. Reproduced with permission from [37]. Copyright 2022 Elsevier.

Regarding dead-end filtration, Hermia was the first person to give a physical derivation of the so-called intermediate blocking law and to provide a single equation linking the four blocking filtration laws for porous media [29]. The mechanisms of fouling associated with each of these laws are illustrated in Figure 3. They are linked by a single equation:

(9) $$\frac{d^{2}t}{dV^{2}}=k_{n}{\left(\frac{dt}{dV}\right)}^{n}$$

where *t* is time, *V* is filtrate volume, *n* is an index characteristic of a particular mode of blocking (see Figure 3) and $k_{n}$ is a constant that is dependent upon the mode of blockage. In [9] it is stated that “in the intermediate blocking mechanism (*n* = 1), the particle size of the feed is the same as the membrane pore size; however the membrane pore is not necessarily plugged by particles”. The second sentence is correct however, as a re-reading of [29] will show, the first sentence of the quote reflects a misunderstanding; no restriction on the ratio of particle size to pore size was given by Hermia [29] other than the implicit one that the particle size is not less than the pore size.

Equation (9) has what to many might seem an odd form. Indeed one of us (RWF) recalls a presenter at the international conference on the Engineering of Membrane Processes in Garmisch-Partenkirchen in May 1992 (a conference which Tony Fane helped organise) having fun with the fact that it seems that time itself is being differentiated twice with respect to a volume. In fact, it is *time from the start of filtration* that is being differentiated and one can readily understand that dt/dV is the reciprocal of AJ. Thus as shown in [38], it can readably be deduced that

(10) $$\frac{-dJ}{dt}=\frac{k_{n}}{A}{\left(AJ\right)}^{3-n}$$

Unlike, Equation (9) there is a physical significance to this question. For dead-end filtration a plot of ln ($\frac{-dJ}{dt})$ versus ln (J) will provide information about the fouling index *n* and hence the mode or modes of fouling. There is a natural tendency to prefer linear relationships of the form “y=mx+c”. Thus a robust methodology for fouling analysis of crossflow systems would ideally involve relationships of this form. For explication of such a methodology—the integral method—see [38] for details of the five steps involved. Here only a few key details are noted. One of the principal advantages of this method over many others is the avoidance of the need to numerically differentiate data [39,40]. Another advantage is that it is a practical method for identifying whether the initial fouling of a membrane is by the same mechanism as the subsequent fouling. Underpinning this methodology is the crossflow equivalent of Equation (10), which following earlier work [30,40], has the form:

(11) $$\frac{-dJ}{dt}=K_{n}J^{2-n}\left(J-J_{R}\right)\;\mathrm{for}\;J>J_{R}$$

where *J* is volumetric flux, *n* is an index characteristic of a particular fouling mechanism and $K_{n}$ is a constant that is dependent upon the mode of blockage and $J_{R}$ is related to the cross-flow removal from the surface of the membrane. Here as in [38] this term is taken to be an empirical parameter that reflects removal term. 

If there are two consecutive phases of fouling, then the value of $J_{R}$ for the second phase will be the steady state flux. The value of $J_{R}$ for the first phase can be expected to be different as in the example given in [38]. Basically the reason is that the surface interactions will be different as for the first phase they involve foulant–membrane interaction and for the second foulant–cake interaction.

Integration of Equation (11) for each of the four values of *n*, followed by rearrangement, leads to four different expressions, however each is of the form “y=mx+c” as given in Table 1 where “*x*” is v/t.

The integral method is a practical method for identifying whether the initial fouling of a membrane is by the same mechanism as the subsequent fouling. Starting with v(t) and J(t) data, the four expressions (Equation (12a–d)) can be evaluated to establish which are the most appropriate. It is unlikely that any one equation adequately fits all the data.

**Figure 4:**
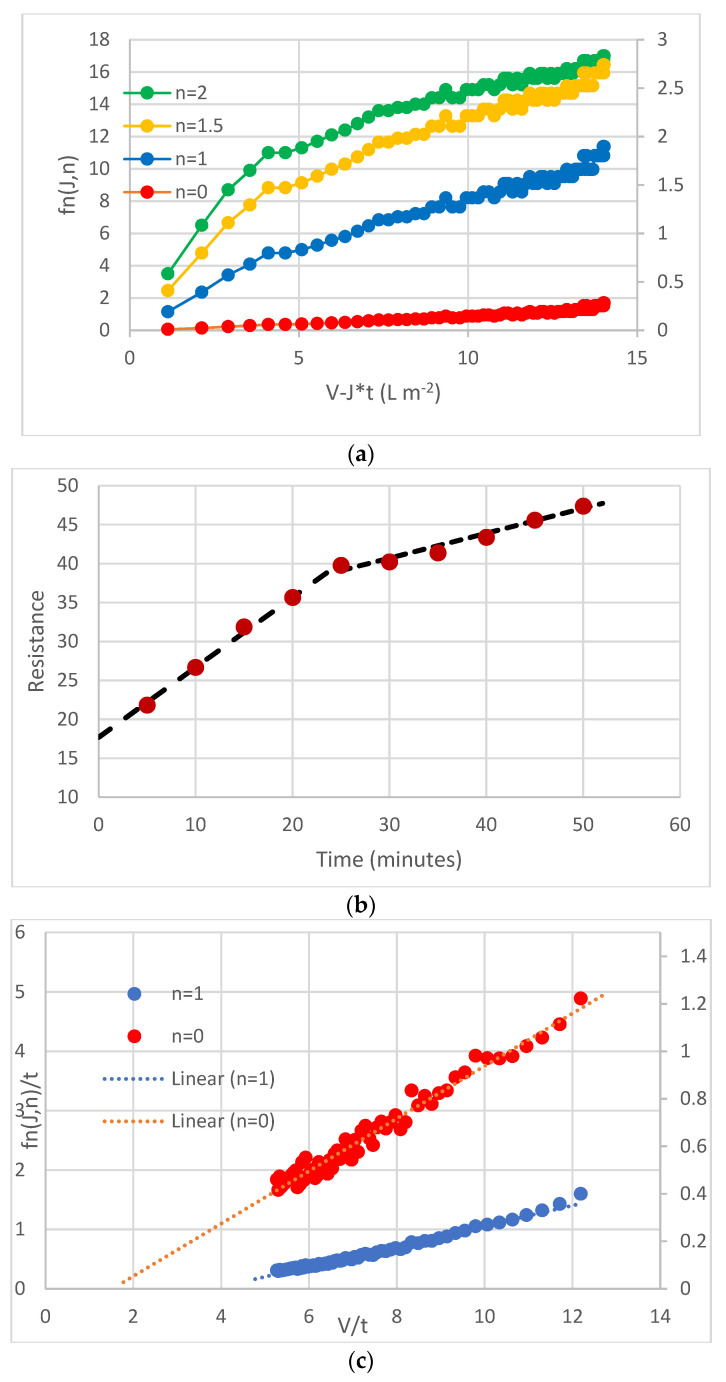

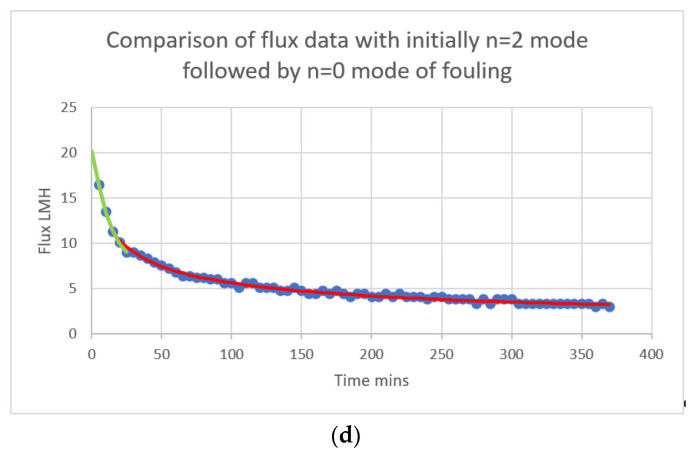
(**a**) Initial plot of data to determine overall possibilities; (**b**) Data used in R(t) format to check on breakpoint from one mode to the next; (**c**) Evaluation of most appropriate mechanism for modelling of second phase; (**d**) final output with data shown in blue.

The plots are likely to reveal a characteristic break as shown in Figure 4a and as found with the data sets checked previously [36,38]. As discussed elsewhere [38] the evaluation of $R_{t}\left(t\right)$ and its derivative with respect to time can illuminate decisions regarding the mode of fouling. Here the principal message from Figure 4b is that the rate of change of resistance is nearly three times greater from 0–25 min as it is from 25–50 min. Once the breakpoint has been identified (here we assume just two phases) the two parts of the J(t) curve are evaluated separately. The main elements of the methodology are detailed elsewhere [38] and are summarized in Figure 4. 

The initial plot of overall possibilities in Figure 4a indicates a breakpoint at 4 L/m^2^ and the existence of a clear breakpoint is confirmed by the resistance–time plot (Figure 4b). Evaluation of the most appropriate mechanism for each phase indicated that the modes *n* = 2 and *n* = 0, for the first and second phases, respectively, gave the best fit to the data. The final output is shown in Figure 4d.

### 3.5. Can One Make Permeate Flux Predictions?

When reading that “the prediction of permeate flux is critical”, one is intrigued as to what input parameters will be used to formulate the prediction; how will the feed solution/suspension be characterized, what will characterize the membrane and how will the hydrodynamics be characterized? Papers elsewhere e.g., [9,41] freely use the word ‘prediction’ as if they will be able to make *a priori* prediction of the permeate flux-time curve. At best such so-called predictions are better described as *a posteriori* deductions. It is not clear to us that the ARIMA models evaluated elsewhere [41] actually convey much new knowledge as a result of the observations and analysis, whereas it is readily appreciated that the integral method of fouling analysis can illuminate the phases of fouling *a posteriori*. However neither phenomenological models nor non-phenomenological models can in general be used to make “predictions” as to predict is to say in advance what will happen in experiments. 

### 3.6. What Differences Are Observed in Operation under Constant Flux?

All of the above discussion has been concerned with constant pressure filtration. The other canonical form of filtration is constant flux filtration, in which the transmembrane pressure (TMP) either stays constant (if there is no fouling) or increases. To illustrate the difference, it is useful to examine the following general membrane equation, in which F represents the net driving force and other terms have their normal meaning (see the nomenclature).

(13) $$J=F/\left[\mu_{p}\left(R_{m}+R_{f}\right)\right]$$

Rearrangement and differentiation with respect to time yields:

(14) $$\frac{dR_{f}}{dt}=\frac{1}{\mu_{p}J}\frac{\partial F}{\partial t}-\frac{F}{\mu_{p}J^{2}}\frac{\partial J}{\partial t}$$

Regarding constant flux operation, the second term on the righthand side is zero. Thus fouling (an increase in $R_{f}$) necessitates an increase in F. Now, historically experiments on constant flux filtration have tended to involve dead-end mode despite the frequent use of constant flux crossflow filtration membrane operation in practical applications. The first study combining experiments and a thorough mathematical analysis for a constant flux crossflow filtration report was recent [37]. Therein a model combining intermediate pore blocking and cake filtration gave the best agreement with the experimental data. Below the threshold flux, an intermediate pore blocking model sufficed, however as permeate flux approached and passed a threshold flux, accurate fitting of the data necessitated use of the combined model. The key finding of that paper is that in constant flux crossflow filtration there can be (at least for some membrane-feed pairs) a finite amount of fouling necessitating just a finite increase in the operating pressure. To many this may seem counter-intuitive, and if one focusses upon the accumulation of a material as a foulant cake (*n* = 0 mode in Table 1) then it is would be counter-intuitive. However, for other modes it is not necessarily counter-intuitive. Indeed when intermediate pore blocking is the only mode of fouling, constant flux crossflow filtration can initially display positive $dR_{f}/dt$ followed by $dR_{f}/dt=0$. A mathematical explanation is given elsewhere [37].

An important caveat with respect to the findings just mentioned is that in that work the membrane was really tight with respect to the suspended particles and/or the droplets. Thus in-pore fouling was physically impossible. Now for those applications, in which the product is recovered in the permeate, there is passage of macromolecules through the membrane, which necessitates very open membranes. Typically these operations are at constant flux and for these applications there have been no reports of a threshold in the build-up of TMP.

**Figure 5:**
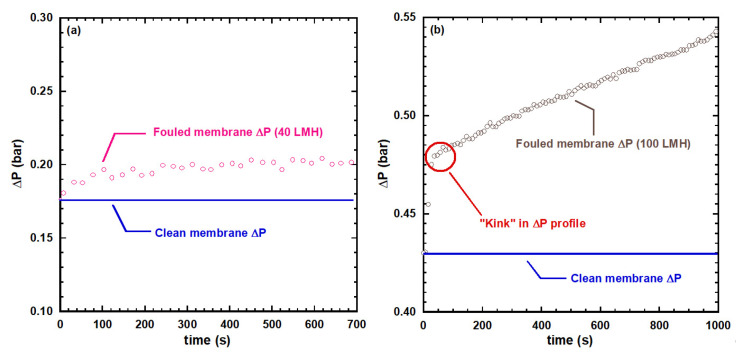
Constant flux filtration conducted with 200 ppm 0.22 µm latex bead suspension below and above the threshold flux (TF). Influence of filtration time on Δ*P* at (**a**) constant flux of 40 LMH (below the TF), and (**b**) constant flux of 100 LMH (above the TF). Reproduced with permission from Elsevier.

The concept of a threshold flux (TF) was first developed ten years ago [42] and it was introduced within the context of constant flux filtration in the water industry. Examining three sets of pilot plant data it was found there was a modest rate of fouling below a certain flux value (which depended upon the water source) however above this value (which varied with the water source) there was a step change in the rate of fouling. The key flux was labelled as a threshold flux (TF) to distinguish it from the concept of critical flux that had been introduced much earlier [30]. The latter is a demarcation of the point below which there is no fouling. When the concept of TF was introduced it separated a region, in which the *rate of fouling* was modest from a region in which the rate of fouling was great, and generally significantly greater. Now in the aforementioned findings [37] regarding a threshold flux in *constant flux* crossflow filtration, in which the ratio of foulant size to membrane pore size was large, the interesting phenomenon illustrated in Figure 5, was observed. Below TF, as shown in Figure 5a, the required TMP rises to a plateau value indicating that there is initially a positive $dR_{f}/dt\;$ however the rate of increase in the foulant resistance then goes to zero. However above TF, as shown in Figure 5b, there is an initial jump in TMP followed by a steady rate of increase. It has yet to be established experimentally whether this behavior can be reproduced with macromolecules. So whilst the findings may be irrelevant when the product is macromolecules in the permeate they are highly relevant to the recovery of water from oily water emulsions and applications involving concentration of a feed. 

## 4. Design

### Is Pilot Plant Evaluation Essential?

Firstly, as noted in a recent paper concerning the microfiltration of skimmed milk, experimental research is essential in the development of any membrane process where the constituents exhibit a “flux paradox” [43]. As they noted, “experimental research on membrane technology is a must to predict proper operating conditions as the gel polarization model underpredicts the flux by 1 to 2 orders of magnitude in colloidal suspensions, hindering the selection of operating conditions that meet the needs of large-scale production”. Furthermore, as they also noted, most of the models that have been proposed as an alternative to the gel polarization model are only valid for laminar flow and/or for some specific particle sizes, however, microfiltration processes in the dairy industry are principally carried out in a turbulent regime [44]. Thus for such fluids membrane models are at best a guide; the process is more complex than, for example, the design of heat exchangers. 

More generally the design of membrane processes involves a number of steps and the claim that permeate flux in UF processes can be predicted ab initio by either phenomenological models or non-phenomenological models is a misrepresentation. One can readily agree that several models are useful “to both describe the reduction in flux and to understand different phenomena involved in membrane filtration” [9] however to go further underplays the crucial role of pilot plant data. The basic steps, as seen by co-authors from academe and industry [45], include at least five steps: (i) Initial membrane screening; (ii) For the selected membranes, experimental evaluation at bench scale of flux and selectivity under different process conditions including various TMPs and concentrations, preferably with realistic hydrodynamic conditions; (iii) After the membranes have been evaluated, an initial process layout can be formulated based on data collected from literature, experiments and simulations. A first economic analysis can then be made to establish whether there is an economic case to move to pilot plant assessment; (iv) The evaluation of actual modules at a pilot unit so as to ensure that the results are transferable to larger scale units. Various cross-flow velocities will need to be examined and for the most promising set of conditions a full run with a number of operating cycles (including cleaning) should be undertaken; (v) The membrane system will be part of an overall process and the potential for synergies should be examined. The work of Krishna Kumar et al. [46] is one example of a food related pilot scale study that coupled an experimental study of the effect of selected operating parameters on flux with cost considerations and the development of models to describe the phenomena observed.

For stage (ii), analysis such as the use of the integral method can aid understanding and help to reduce the number of experiments. Furthermore, understanding the role of elements of modules such as spacers is important. Here again Tony Fane was involved [47]. Stage (iv) may reveal that the hydrodynamic conditions at the pilot scale lead to a poorer performance than that found at the laboratory scale. Sometimes the mass transfer conditions at the module scale are noticeably inferior to those that had appertained at the laboratory scale [48]. As noted previously in the paper, fouling time scales range from minutes to hours, however, as found in practice there is also potentially another timescale of days. This reinforces the need to work at the pilot scale. An important note of detail is that if the feedstock is recycled during the evaluation period then the amount of key foulant per unit area of membrane area may be very different from that of the proposed full-scale plant. Consideration needs to be given to foulant load as well as time of operation. Following pilot scale evaluation, a full-scale unit can be simulated and optimized, although extrapolation outside of the parameter space covered by the pilot tests is to be avoided. There could be a role for non-phenomenological models at this stage, however we are not aware of such work.

## 5. Concluding Remarks

Whilst the definition of concentration polarization (CP) as the rapid emergence of concentration gradients at a membrane/solution interface resulting from selective transfer through the membrane will be readily accepted by almost the whole membrane community, there is often a confusion between the effects of CP itself (such as increased osmotic pressure at the interface) and fouling that may arise as a result of the increased concentration at the interface. CP is distinguishable from fouling in at least two ways: (1) the timescale for the establishment of the effect; and (2) the state of the molecules involved. Regarding the first, CP will normally be established in less than a minute although fouling can continue over timescales of varying orders of magnitude. Crucially, CP involves molecules in solution whereas fouling by macromolecules involves molecules no longer in solution.

Secondly, care in the definition of terms can often tease out subtleties that are important. There are two paradigmatic approaches for modelling flux, one uses the overall driving force (in which case allowance for osmotic effects are expressed as additional resistances) and the other uses the net driving force across the separating layer or fouled separating layer, although often the two are unfortunately comingled. It is wrong to include a resistance to the concentration polarization layer, $R_{cp}$ in the denominator of the flux equation, whilst simultaneously including Δπ in the numerator. One needs to choose whether one is establishing an equation across the membrane itself, or from the bulk feed on one side to the permeate on the other.

Whilst various approaches to the categorization of fouling models can be debated, the use of dead-end models for the analysis of fouling in crossflow systems is to be avoided. The attraction of taking the ‘short-cut’ was identified as the easy way relationships of the form “y=mx+c” can be used. For the analysis of crossflow data the integral method of fouling analysis is recommended. In addition to determining, in a clear-cut manner, the point at which there is a switch from one mode to another, the robust methodology yields characteristic J(t) equation for each mode that are an excellent fit to the data.

## Figures and Tables

**Table 1:** Tabulated expressions permitting facile crossflow fouling analysis.

Complete pore blocking with allowance for crossflow removal	n=2	$\left(J_{0}-J\right)/t=K_{n}v/t-K_{n}J_{R}$	(12a)
Pore filling mechanism ^(1)^	n=1.5	$\left(J_{0}^{0.5}-J^{0.5}\right)/t=K_{n}v/t-K_{n}J_{R}$	(12b)
Intermediate pore blocking with allowance for crossflow removal	n=1	$\ln(J_{0}/J)/t=K_{n}v/t-K_{n}J_{R}$	(12c)
Cake formation with allowance for crossflow removal	n=0	$\left(1/J-1/J_{0}\right)/t=K_{n}v/t-K_{n}J_{R}$	(12d)

^(1)^ For this fouling mechanism, the value of $J_{R}$ is expected to be zero.

## Data Availability

Not applicable.

## Abbreviations and Nomenclature

ARIMA	Autoregressive integrated moving average
CP	Concentration polarisation
ln	natural logarithm
Pe	Boundary layer Peclet number
TMP	Transmembrane Pressure
UF	Ultrafiltration
	
*A*	area of membrane
*C*	concentration
*D*	diffusivity
*F*	net driving force
*J* or *J*(*t*)	volumetric flux
*K_n_*	constant dependent upon the mode of blockage (Equation (11))
*k*	boundary layer mass transfer coefficient (=D/δ)
*k_n_*	constant dependent upon the mode of blockage (Equation (9))
*n*	index characteristic of a particular mode of blocking
*R_f_*	foulant resistance (m^−1^)
*R_m_*	hydraulic resistance of the membrane (m^–1^)
*R_t_*	overall resistance (m^−1^)
*t*	time
*V*	filtrate volume
*v*	specific volume (=V/A)
δ	thickness of mass transfer boundary layer
*μ*	viscosity
$\mu_{p}$	viscosity of the permeate
Δ*P*	transmembrane pressure difference
Δπ	osmotic pressure difference across the membrane ($=\pi_{m}-\pi_{p})$

## Subscripts

0	initial
b	bulk
cp	concentration polarisation
gel	gel
lim	limit
m	upstream membrane surface
p	permeate
*R*	removal term
